# Overexpression of let‐7d explains down‐regulated KDM3A and ENO2 in the pathogenesis of preeclampsia

**DOI:** 10.1111/jcmm.16299

**Published:** 2021-08-05

**Authors:** Qian Xu, Yonghui Song, Lili Lu

**Affiliations:** ^1^ Department of Obstetrics Linyi People’s Hospital Linyi China

**Keywords:** ENO2, KDM3A, Let‐7d, methylation, Pre‐eclampsia, trophoblast

## Abstract

Pre‐eclampsia (PE) is the leading cause of maternal death; however, the causative molecular basis remains largely unknown. Recent studies have revealed the important role microRNAs (miRNAs) play in PE. We aimed to explore the effects of let‐7d on trophoblast proliferation, migration, invasion and apoptosis in PE and its underlying mechanism. Placental tissues were collected from PE patients and healthy pregnant women, and it was found that let‐7d expression was increased, while KDM3A and ENO2 expression was decreased in PE tissues and cells. Bioinformatics analysis indicated the interaction among let‐7d, KDM3A and ENO2, confirmed by dual luciferase reporter gene assay; ChIP experiment identified methylated modification to ENO2 by KDM3A. With gain‐ and loss‐function method, silencing of let‐7d increased KDM3A expression and enhanced the binding between KDM3A and ENO2. Furthermore, overexpression of let‐7d suppressed cell proliferation, migration and invasion of trophoblasts, and induced apoptosis of trophoblasts, while these capacities were restored upon additional treatment of overexpressed ENO2. PE rat models were established to explore the effects of let‐7d and ENO2 on PE in vivo. The results established that the silencing of let‐7d alleviated the tissue injury and PE‐related symptoms when reducing urine protein, TUNEL‐positive cells and increasing ENO2, and KDM3A expression in rats. Cumulatively, let‐7d suppressed cell progression of trophoblasts, and induced apoptosis through the down‐regulation of KDM3A to promote ENO2 methylation, thereby promoting progression of PE. Such an epigenetic network of let‐7d, KDM3A and ENO2 in the pathogenesis of PE might provide novel insight into targeted therapy against this disorder.

## 1 INTRODUCTION

Pre‐eclampsia (PE) is known as a placentally induced hypertensive disorder during pregnancy, which is a leading cause of maternal mortality and morbidity.^1^ Statistics reveal high mortality rate of PE in low‐income countries with insufficient adequate prenatal and obstetric care.^2^ This disease is often accompanied by the hypertension, proteinuria, kidney and liver impairment.^3^ Currently, there has been no reliable prediction method in clinical practice, and the only effective treatment is to deliver of placenta and infant, usually prematurely, which in turn increases the risk of foetal complications.^4^ Additionally, PE has been reported to correlate with superficial trophoblastic infiltration and abnormal placental formation which endangers persistent placental hypoxia and the secretion of multiple mediators into the maternal circulation.^5^ Therefore, it is imperative to uncover the molecular mechanisms of PE and identify effective biomarkers during the development and progression of PE in order to provide potential targets for the prevention and treatment of PE.

MicroRNAs (miRNAs) are small non‐coding RNAs involved in regulating gene expressions.^6^ MiRNAs are implicated in trophoblast migration and invasion, serving as potential biomarkers in PE.^7^ Varying evidence has established that let‐7d is highly conserved in miRNAs family across animal species, and it could potentially regulate several cellular responses in human cancers.^8^ Additionally, let‐7d has been demonstrated to play an important role in PE,^9^ but the exact role of let‐7d in trophoblast dysfunction, a known hallmark of the pathogenesis of PE, remains elusive. Moreover, microarray analysis in the current study's preliminary experiments has shown that let‐7d could target and modulate lysine demethylase 3A (KDM3A). KDM3A, also known as JMJD1A or JHDM2A, is of great significance for gene regulation in various biological activities, such as tumour progression.^9^ Furthermore, KDM3A acts as a regulator in key downstream events in the development of invasive trophoblast lineage, especially those necessary for trophoblast‐directed uterine spiral artery reconstruction where KDM3A facilitates critical adaptations to environmental changes.^10^ The enzyme enolase 2 (ENO2) has also been established to be involved in PE.^11^ ENO2, known as neuron‐specific enolase (NSE), consists of two enolase isoenzymes, γγ and αγ, and is primarily released by mature neurons and cells of neuronal origin.^12^ Previously conducted studies have shown that hypoxia‐inducible transcription factors (HIF) can bind to the HRES site of KDM3A to stimulate the transcription of KDM3A.^13^, ^14^ KDM3A can regulate the downstream target genes through epigenetic modification. Additionally, KDM3A regulates MMP12 and controls trophoblast invasion and reconstruction of uterine spiral arterioles, contributing to the occurrence of placental diseases.^10^ The RNA‐Seq technology once indicated that 293 PE‐related genes were aberrantly expressed and PE‐related key genes in PE, including the ADM, SERPINF1 and ENO2,^11^, ^15^, ^16^ where ENO2 was closely related to glycolysis/gluconeogenesis and HIF‐1 signalling pathway in PE. Other studies have shown that both KDM3A and ENO2 are abnormally expressed in PE, and are closely related to the HIF signalling pathway. KDM3A may be delivered to its target genes through HIF transcription factors, regulating the expression of downstream target genes of KDM3A.^10^ Therefore, KDM3A may be potentially involved in the development of PE through methylating ENO2 expression. The current study hypothesized that let‐7d regulates ENO2 via KDM3A in the trophoblast proliferation, migration and invasion, and enhanced apoptosis in PE. Thus, the aim of the present study was to investigate the effects of let‐7d targeting KDM3A in relation to its effect on the cellular processes in PE combined with regulation of KDM3A.

## 2 METHODS

### 2.1 Ethics statement

The ethical approval for the use of human specimens was obtained from the Research Ethics Committee of Linyi People's Hospital. Signed written informed consents were obtained from all the patients included in the study. The experiments were conducted in accordance with the ethical standards formulated in the *Helsinki Declaration*. All animal experiments were conducted in strict accordance with the Guide for the Care and Use of Laboratory animals published by the US National Institutes of Health (NIH). The animal experiments were approved by the Ethics Committee of Linyi People's Hospital. Great efforts were made to minimize the number of animals used in the experiments and their suffering.

### 2.2 Study subjects

In the current study, 73 pregnant women were recruited from June 2017 to June 2018 for prenatal examination and delivery in the Department of Obstetrics in Linyi People's Hospital; these included 35 patients with PE and 38 healthy pregnant women, according to their symptoms and signs.^17^ All enrolled women were carrying singleton pregnancies. Except PE, there were no other medical and surgical complications before and after pregnancy with the patients clinical data presented in Table S1. Subsequent to vaginal delivery or caesarean section, placental tissue blocks of about 1 cm × 1 cm × 1 cm were collected under an aseptic environment, all around placental foetal surface and 2 cm from the root of the umbilical cord. The calcified plaques were avoided and the placental samples were eventually divided into two groups within 10 min of the delivery of the placenta. The placental tissues were rinsed twice with normal saline and dried with gauze. One group of placental tissues was promptly put into a −80°C freezer, which was used for molecular biological detection, while another group was fixed in 4% paraformaldehyde for immunohistochemical staining.

### 2.3 Haematoxylin‐eosin (HE) staining

The paraffin‐embedded placental tissues were cut into serial slices of 5 μm thickness. Subsequently, the slices were dewaxed in xylene, hydrated with gradient alcohol, stained with haematoxylin solution for the duration of 15 min, and then stained with eosin solution for the duration of 30 min. Following the routine steps of dehydration and clearing, the slices were sealed with neutral balsam and observed under a microscope.

### 2.4 Immunohistochemical staining

The paraffin‐embedded placental tissues were cut into serial slices of 5 μm thickness, which were dewaxed in xylene, and hydrated with gradient alcohol. The sections were then incubated for the duration of 10 min at room temperature in 100% methanol and 3% H_2_O_2_ to quench the endogenous peroxidase activity. Sections were subsequently blocked with 10% BSA, and then incubated with a primary antibody against KDM3A (1:500, ab251059; Abcam Inc) overnight at the controlled temperature of 4°C, which was followed by staining with ABC immunohistochemical staining. The samples were then stained with diaminobenzidine (DAB), dehydrated, cleared, and then embedded in paraffin. The micrographs were taken under a microscope, and cells with clear brownish yellow staining in the cytoplasm were identified as the positive cells. Additionally, phosphate buffered saline (PBS) and normal goat serum were used to replace the primary antibodies for immunohistochemical staining to establish the control group, while the other steps remained the same as the experimental group.

### 2.5 Cell treatment and transfection

Human choriocarcinoma cell lines BeWo and JEG‐3 (American Type Culture Centre) were placed in a previously prepared Dulbecco's modified Eagle's medium (DMEM) which consisted of 10% foetal bovine serum [FBS, Gibco], 100 U/mL penicillin, and 100 μg/mL streptomycin [Lonza]) at 37℃ and 5% CO_2_. Subsequently, the cells were transfected with expression vectors containing KDM3A (plasmid provided by the Shanghai Sangon Biotech), short hairpin RNA (shRNA) targeting KDM3A, exogenous let‐7d mimic, exogenous let‐7d inhibitor, expression vectors containing ENO2, and shRNA targeting ENO2 plasmids. The cells were transfected in accordance to the instructions of Lipofectamine 2000 (Invitrogen).

### 2.6 Colony formation assay

The BeWo and JEG3 cells of each group were collected 24 hours subsequent to the trypsin detachment and transfection. Following the counting of single cell suspension, the cells were diluted, inoculated into six‐well plates of 1,000 cells per well, and cultured in 2 ml of medium at 37℃ and 5% CO_2_ for the duration of 10‐12 days. The culture was terminated when the colonies appeared. Subsequently, the cells were fixed with 4% paraformaldehyde and stained with crystal violet solution for the duration of 15 min. Following the above mentioned steps, the staining solution was washed out with ultra‐pure water and dried upside down on the operating table. The colonies containing over 50 cells were counted under a microscope. The number of cell colonies was counted with naked eyes in an inverted culture dish.

### 2.7 3‐(4,5‐dimethylthiazol‐2‐yl)‐2,5‐diphenyltetrazolium bromide (MTT) assay

Subsequent to the trypsin detachment, the transfected BeWo and JEG‐3 cells were added to DMEM for logarithmic growth, and the cells were suspended and counted under an inverted microscope. The cell concentration was adjusted to 5 × 10^4^ cells/mL, and seeded into a 96‐well plate. Following incubation for duration of 48 h, cells were cultured with 10 μL MTT for 4 h at the temperature of 37℃. Furthermore, subsequent to the removal of the supernatant, dimethylsulphoxide (DMSO) (150 µL/well) was added to dissolve MTT. Cell viability was determined by measuring the optical density (OD) at the wavelength of 570 nm.

### 2.8 Transwell invasion assay

The box containing the transwell chamber was heated and placed in the 24‐well plate. The medium was also preheated in the incubator. For cell invasion experiment, the transwell chamber was added with the mixture of Matrigel (BD Biosciences) and culture medium at a ratio of 1:4, and further incubated at 37℃ for 4 h to solidify the Matrigel gel. Subsequently, 0.5 ml of medium was added into the apical and basolateral chambers for hydration in the incubator for the duration of 2 h. Following the detachment, a cell suspension (5 × 10^4^ cells/ mL) was prepared. After this, 0.5 ml complete medium was added, the hydrated chamber was transferred to the 24‐well plate, and then 0.1 ml diluted cell suspension was further added. The resultant cells were then incubated for 24 h, which was followed by the removal of the liquid in apical and basolateral chamber. The cells on the upper surface of the basal membrane were removed using a sterile cotton swab, washed three times with PBS, fixed in precooled paraformaldehyde for the duration of 30 min, stained with crystal violet for the duration of 10 min, and finally rinsed with running water. The dried crystal violet was observed under the inverted microscope (Olympus).

### 2.9 Flow cytometry

Subsequent to the detachment with trypsin without ethylenediaminetetraacetic acid (EDTA) for 15 min, the cells were washed with precooled PBS. The cell apoptosis was quantified by flow cytometry using a commercially available Annexin‐V‐FITC apoptosis detection kit (556 547, Shanghai SOLJA Technology Co., Ltd.) in accordance to the manufacturer's instructions. Furthermore, Annexin‐V‐FITC/PI was prepared at a ratio of 1:2: 50. The resultant cells were counted and re‐suspended at a density of 1 × 10^6^ cells/ml. They were then incubated at room temperature for 15 min, washed with pre‐cooled PBS, and mixed again. Fluorescence was initiated by excitation at 488 nm and was measured by emission filters at 515 nm (FITC) and 620 nm (PI) so as to detect cell apoptosis.

### 2.10 RNA isolation and quantification

The reverse transcription quantitative polymerase chain reaction (RT‐qPCR) was performed to determine the levels of miRNA and mRNA in the placental tissues and trophoblasts. The total RNA was extracted from the obtained tissues and cells using the RNeasy Mini Kit (Qiagen). The reverse transcription of mRNA into complementary DNA (cDNA) was performed using the Reverse transcription Kit (RR047A, Takara Bio Inc, Otsu). Subsequently, for miRNA detection, the total RNA was reverse transcribed into cDNA using the miRNA First Strand cDNA Synthesis (Tailing Reaction) kit (B532453‐0020, Shanghai Sangon Biotech). Furthermore, the RT‐qPCR analysis was conducted using the SYBR® Premix Ex TaqTM II (Perfect Real Time) kit (DRR081) on the ABI7500 quantitative PCR instrument (ABI). The primers used are listed in Table 1.

**Table 1:** Primer sequences for RT‐qPCR

Target		Sequences (5’‐3’)
miR‐let‐7d	Forward	5'‐CAGAGGTAGTAGGTTGC −3'
	Reverse	5'‐GCGCGTGAGCAGGCTGGAGAAATTAACCACGCGCAACTAT‐3'
U6	Forward	5'‐ACGCAAATTCGTGAAGCGTT −3'
	Reverse	Universal primer from TAKARA
KDM3A	Forward	5'‐CTGAGATTCCTGAGCAAGTTATTC −3'
	Reverse	5'‐AGCCGAAGACTGTTTACATCC −3'
ENO2	Forward	5'‐ GCG ACC AGA AGG GTA TTG AC −3'
	Reverse	5'‐ AGC CCC ACT CGT TCT TAG TTC −3'
GAPDH	Forward	5'‐AATGGGCAGCCGTTAGGAAA −3'
	Reverse	5'‐GCGCCCAATACGACCAAATC −3'

### 2.11 Western blot analysis

The total protein was extracted using RIPA lysis containing PMSF, incubated on ice for the duration of 30 min, and subsequently centrifuged at 12 000 r/min at 4℃ for 10 min. The resultant supernatant was collected, and its protein concentration was measured using the BCA Kit (23225, Pierce), and adjusted with deionized water. The protein (50 µg/well) was then separated by 10% sodium dodecyl sulphate polyacrylamide gel (SDS‐PAGE) (P0012A, Beyotime Biotechnology, Co., Ltd.) at a constant voltage of 80 V for the duration of 2 h. The samples were subsequently transferred onto a polyvinylidene fluoride (PVDF) membrane at a constant voltage of 110 V for 2 h. The membrane was then blocked with Tris‐buffered saline and Tween‐20 (TBST) containing 5% non‐fat milk for the duration of 2 h. Following the removal of sealing solution and washing with TBST, the membrane was incubated with the primary rabbit anti‐human antibodies at 4°C overnight, including KDM3A (1:1000, ab80598), ENO2 (1:1000, ab222514), MMP‐9 (1:1000, ab73734), Vimentin (1:1000, ab137321) and β‐actin (1:1000, ab179467). All the primary antibodies were purchased from Abcam Inc The membrane was then washed with PBST (PBS containing 0.1% Tween‐20) three times, for 10 min each time and incubated with horseradish peroxidase (HRP)‐conjugated goat anti‐mouse immunoglobulin G (IgG) secondary antibody (1:5000, Beijing Zhongshan Biotechnology, Co., Ltd.), which was followed by washing with TBST. Subsequently, the moisture on the surface of the membrane was absorbed using a filter paper, and the membrane was flatted on the loading platform. The A and B developer was mixed at a ratio of 1:1 and dropped on the membrane, which was developed using the chemiluminescence method. The relative expression of protein, expressed as the grey value of corresponding protein bands/the grey value of β‐actin protein bands, was analysed employing the Quantity One v4.6.2 software.

### 2.12 Dual luciferase reporter gene assay

The targeting correlation between let‐7d and KDM3A was verified using online prediction software and the dual luciferase reporter gene assay. The binding site between let‐7d and KDM3A was analysed to obtain the fragment sequence containing the action site. The 3′‐untranslated region (3′‐UTR) sequence of KDM3A with wild type (WT) or mutated (MUT) let‐7d binding site was cloned into the target sequence of psiCheck2 plasmid downstream of luciferase reporter gene (KDM3A‐WT and KDM3A‐MUT). Subsequently, the luciferase report vectors were co‐transfected into cells with NC mimic, let‐7d mimic, inhibitor‐NC and let‐7d inhibitor, and the resultant luciferase activity was measured using the luciferase test kit (Promega). Following incubation for 48 h, the cells were split in 1 × passive cleavage, and the firefly luciferase activity was measured employing the Dual Luciferase Reporter Assay System (Promega) with renilla luciferases as internal reference.

### 2.13 ChIP assay

The BeWo cells were fixed with 1% formaldehyde at room temperature for the duration of 10 min to crosslink DNA and protein. The cells were then sonicated to fragments with 10 s each time at the interval of 10 s for 15 cycles. Subsequently, the resultant cells were centrifuged at 13 000 rpm at 4℃ to obtain the supernatant, which was then divided into the two tubes. Additionally, the membrane was incubated with the negative control antibody rabbit anti‐IgG (ab109489, 1:100, Abcam) and the target protein specific antibody mouse anti‐KDM3A (ab91252, 1:100, Abcam) or H3K27me3 (ab6002, 1:100, Abcam), respectively, at 4℃ for overnight. Furthermore, the endogenous DNA‐protein complex was precipitated by Protein Agarose/Sepharose, and the supernatant was removed subsequent to centrifugation. The non‐specific complex was washed, the cross‐linking was removed overnight at the controlled temperature of 65℃, and the DNA fragments were extracted and purified using phenol/chloroform. The enrichment of ENO2 promoter fragments combined with KDM3A and H3K27me3 was examined by the specific primers of ENO2 promoter fragments.

### 2.14 Rat model of PE

Sprague‐Dawley (SD) rats (5‐6 weeks old, 210‐250 g) were obtained from the Hunan SJA Laboratory Animal Co., Ltd. The female rats were copulated with weight‐matched male rats at a female: male ratio of 2:1 during the oestrus period, and the vaginal secretions were microscopically examined every morning. The time with presence of sperms was identified as the 0th gestational day.

Twenty‐four pregnant female SD rats subsequently received subcutaneous injections of N‐nitro‐L‐arginine methyl ester (L‐NAME, Sigma) at a concentration of 125 mg/kg/day beginning on the 12th gestational day for 7 consequent days to establish a PE rat model; whereas, the rats receiving no treatment were taken as controls. On the 12th gestational day, the abdomen of the rat was dissected under anaesthesia and the uterus injected with antagomir‐NC + sh‐NC, antagolet‐7d + sh‐NC and antagolet‐7d + sh‐ENO2, respectively. Controlled rats received subcutaneous injection of normal saline with equal volume of L‐NAME on the 12th gestational day for 7 days.

### 2.15 Blood pressure measurement and urine analysis

The systolic blood pressure (SBP) of each rat was determined using the non‐invasive tail‐cuff method and a BP‐2000 Blood Pressure Analysis System 267 (Visitech Systems, Inc). Each rat was preheated to 38°C for the duration of 5 min before each measurement. Additionally, the 24‐hour urine output of each animal was collected. Proteinuria was detected in the urine samples using the CBB kits (Jiancheng Institute of Biotechnology).

### 2.16 Terminal deoxynucleotidyl transferase mediated dUTP nick‐end labelling (TUNEL)

Apoptosis in the human placental tissues was detected using the TUNEL staining in accordance to the manufacturer's instructions of In Situ Cell Death Detection kit (Roche Diagnostics, GmbH).

### 2.17 Statistical analysis

All data obtained were analysed using the SPSS 21.0 software (IBM Corp.). The continuous data were described as mean ± standard deviation. The comparison between the two groups was performed using an unpaired t test. Whereas, the data comparisons between multiple groups were performed by the one‐way analysis of variance (ANOVA) with Tukey's test. The correlation among let‐7d, KDM3A and ENO2 was analysed using Pearson correlation analysis. A *P* value < 0.05 indicated statistically significant difference.

## 3 RESULTS

### 3.1 KDM3A was poorly expressed in placental tissues in PE patients

**FIGURE 1:**
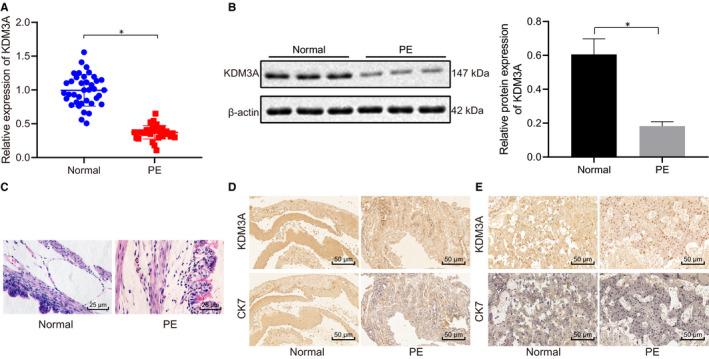
KDM3A was decreased in placental tissues of PE patients. A, mRNA levels of KDM3A in placental tissues in PE patients and healthy pregnant women were determined using RT‐qPCR, normalized to GAPDH. B, Protein levels of KDM3A in placental tissues in PE patients and healthy pregnant women were determined using Western blot analysis, normalized to β‐actin. C, Representative images (scale bar = 25 μm) of pathological characteristics of placental tissues by HE staining. D, KDM3A expression and CK7 protein in placental tissues in PE patients and healthy pregnant women were detected by immunohistochemical staining (scale bar = 50 μm). E, KDM3A and endothelial cell marker CD31 protein and co‐localization in placental villus in PE patients and healthy pregnant women were detected by immunohistochemical staining (scale bar = 50 μm). * (relative to the cells from healthy pregnant women) indicate *P* < .05 by unpaired t test

The RT‐qPCR and Western blot analyses of the placental tissues showed that the KDM3A gene expression and protein levels were decreased in placental tissues obtained from PE patients in comparison to those obtained from healthy pregnant women (*P* < .05) (Figure 1A, B). Additionally, the HE staining of the placental tissues demonstrated that the trophoblast nuclei of the placental tissues of healthy pregnant women were arranged in order with a small number of red cells in the lumen of villi capillaries and fibrinoid necrosis around villi. Whereas, the placental tissues of PE patients displayed underdeveloped placental villi, varying thickness of the matrix, obvious interstitial oedema, disorderly arranged syncytial trophoblasts, and a decreased number of blood vessels (Figure 1C). Furthermore, the micrographs of immunohistochemical staining revealed that positive KDM3A and trophoblast biomarker CK7 protein were present in trophoblasts with co‐localization (Figure 1D). The immunohistochemical detection analysis additionally, identified the co‐localization of KDM3A protein and endothelial cell molecular marker CD31 protein in normal villi and PE villi (Figure 1E). In healthy pregnant women, there were numerous brownish yellow KDM3A granules found in the placenta, which were mainly distributed in the trophoblasts and vascular endothelial cells. Whereas, the placentas of PE patients displayed lighter staining of the placenta, thinner KDM3A particles and a decreased number of positive cells. Therefore, the present study speculated that KDM3A expression was reduced in the placental tissues of PE patients.

### 3.2 KDM3A promoted trophoblast proliferation, migration and invasion, and inhibited apoptosis in vitro

**FIGURE 2:**
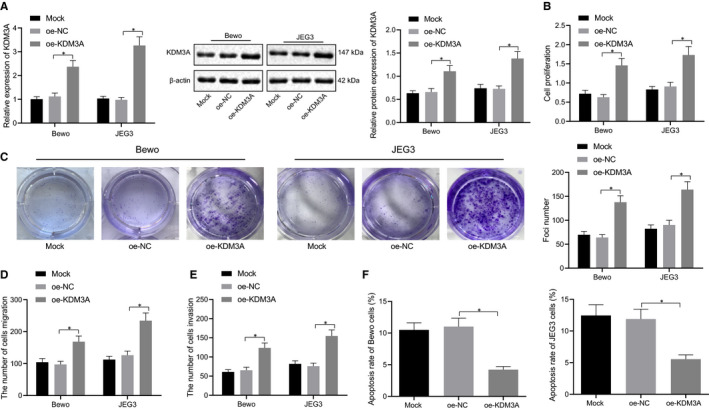
KDM3A promotes proliferation, migration, invasion of trophoblasts and suppresses apoptosis in vitro. BeWo and JEG3 cells were treated with expression vectors containing KDM3A. A, mRNA and protein levels of KDM3A in BeWo and JEG3 cells were determined using RT‐qPCR and Western blot analysis. B, Proliferation of BeWo and JEG3 cells was detected by MTT assay. C, Clone formation of BeWo and JEG3 cells was detected by colony formation assay. D, Migration of BeWo and JEG3 cells was detected by Transwell assay. E, Invasion of BeWo and JEG3 cells was detected by Transwell assay. F, Apoptosis of BeWo and JEG3 cells was detected by Flow cytometry. * (relative to BeWo and JEG3 cells treated with oe‐NC) indicate *P* < .05 by one‐way ANOVA/Tukey's test

With the aim to investigate the effect of KDM3A on cell proliferation, migration and apoptosis of human trophoblasts, BeWo and JEG3 cells were treated with expression vectors containing KDM3A, and their mimicking activity was confirmed by the RT‐qPCR and the Western blot analyses (Figure 2A). Additionally, the MTT assay (Figure 2B), the colony formation assay (Figure 2C), the Transwell assay (Figure 2D, E) and the flow cytometry analysis (Figure 2F) revealed that the trophoblasts with KDM3A overexpression exhibited increased cell proliferation, migration, invasion, increased number of colony formation, and inhibited apoptosis (*P* < .05). The aforementioned findings established that KDM3A promoted cell proliferation, migration and invasion, and inhibited the apoptosis of trophoblasts in cell lines.

### 3.3 Let‐7d could target and negatively regulate KDM3A

**FIGURE 3:**
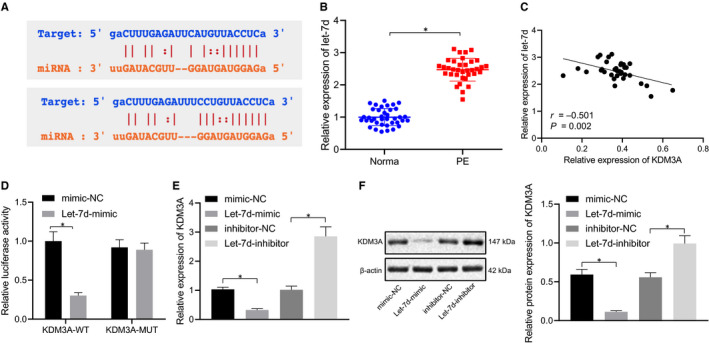
Let‐7d could target and inhibit KDM3A expression. A, Target correlation between let‐7d and KDM3A was predicted using StarBase website (http://starbase.sysu.edu.cn/starbase2/). B, Let‐7d expression in placental tissues in PE patients and healthy pregnant women was determined using RT‐qPCR, normalized to U6. C, Correlation between let‐7d and KDM3A in placental tissues in PE patients was analysed by Pearson correlation analysis. D, Luciferase activity of KDM3A‐WT 3’UTR and KDM3A‐MUT 3’UTR was detected using dual luciferase reporter gene assay. BeWo cells were treated with let‐7d mimic and controls. E, KDM3A mRNA expression in BeWo cells was determined using RT‐qPCR. F, KDM3A protein expression in BeWo cells determined using Western blot analysis, normalized to β‐actin. * (relative to healthy pregnant women or BeWo cells treated with mimic‐NC or exogenous inhibitor‐NC) indicate *P* < .05 by unpaired t test. The correlation between let‐7d and KDM3A was analysed using Pearson analysis

The forward regulatory molecule of KDM3A in the progression of PE was determined using the StarBase website to predict the miRNA that targeted KDM3A in human and rats (Figure 3A). It was revealed that in comparison to the healthy pregnant women, the let‐7d expression was elevated in placental tissues of the PE patients (*P* < .05) (Figure 3B), and also that the let‐7d had a negative correlation with KDM3A in the placental tissues of PE patients (Figure 3C). Furthermore, the dual luciferase reporter gene assay was employed to further verify whether let‐7d targeted KDM3A. The results of the analysis proved that the luciferase activity of KDM3A‐WT 3’UTR was significantly inhibited by let‐7d (*P* < .05); whereas, no statistical difference was found in KDM3A‐MUT 3’‐UTR (*P* > .05) (Figure 3D), which suggested that let‐7d could target KDM3A. Additionally, in order to confirm the interrelationship between them, the BeWo cells were transfected with exogenous let‐7d mimic and exogenous let‐7d inhibitor. The RT‐qPCR and Western blot analyses revealed that the BeWo cells transfected with the let‐7d mimic exhibited decreased expression of KDM3A, while the silencing of let‐7d resulted in the elevation of KDM3A (*P* < .05) (Figure 3E, F). Moreover, it was found that let‐7d could target and negatively regulate KDM3A. The obtained data implied that KDM3A was a target gene of let‐7d.

### 3.4 Let‐7d suppressed the proliferation, migration, invasion of trophoblasts and enhanced apoptosis by down‐regulating KDM3A in vitro

**FIGURE 4:**
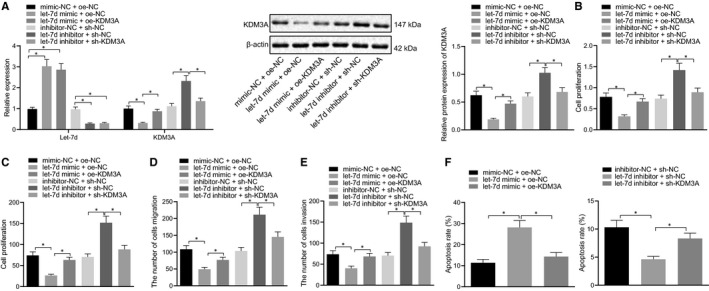
Let‐7d suppressed proliferation, migration, and invasion of trophoblasts and enhanced apoptosis by down‐regulating KDM3A. BeWo cells were treated with exogenous let‐7d mimic, exogenous let‐7d inhibitor, expression vectors containing KDM3A and short hairpin RNA targeting KDM3A. A, Expression of let‐7d, KDM3A and ENO2 in BeWo cells was determined using RT‐qPCR and Western blot analysis. B, Proliferation of BeWo cells was detected by MTT assay. C, Clone formation of BeWo cells was detected by colony formation assay. D, Migration of BeWo cells was detected by Transwell assay. E, Invasion of BeWo cells was detected by Transwell assay. F, Apoptosis of BeWo cells was detected by Flow cytometry. * (relative to BeWo cells treated with mimic‐NC + oe‐NC, let‐7d mimic + oe‐NC, inhibitor‐NC + sh‐NC or let‐7d inhibitor + sh‐NC) indicate *P* < .05 by one‐way ANOVA/Tukey's test

To understand the role of let‐7d and KDM3A in the progression of trophoblasts, the BeWo cells were transfected with exogenous let‐7d mimic, exogenous let‐7d inhibitor, expression vectors containing KDM3A, and shRNA targeting KDM3A. The RT‐qPCR and Western blot analyses detected the let‐7d and KDM3A expression in the BeWoll cells and confirmed the transfection efficiency (Figure 4A). Additionally, the MTT assay (Figure 4B), the colony formation assay (Figure 4C), the Transwell assay (Figure 4D, E), and the flow cytometry analysis (Figure 4F) established that following the overexpression of let‐7d, the cell proliferation, migration, and invasion were inhibited, the number of clone formations was reduced, and the apoptosis was promoted in the BeWo cells transfected with exogenous let‐7d mimic, or exogenous let‐7d inhibitor and the shRNA targeting KDM3A. Meanwhile, the trends were found to be reversed in the BeWo cells treated with exogenous let‐7d inhibitor or exogenous let‐7d mimic and expression vectors containing KDM3A. Hence, the current study concluded that let‐7d could suppress the proliferation, migration, and invasion of trophoblasts, and facilitate the apoptosis through the down‐regulation of KDM3A.

### 3.5 Let‐7d promoted ENO2 histone methylation to inhibit ENO2 expression via inhibiting KDM3A pathway

**FIGURE 5:**
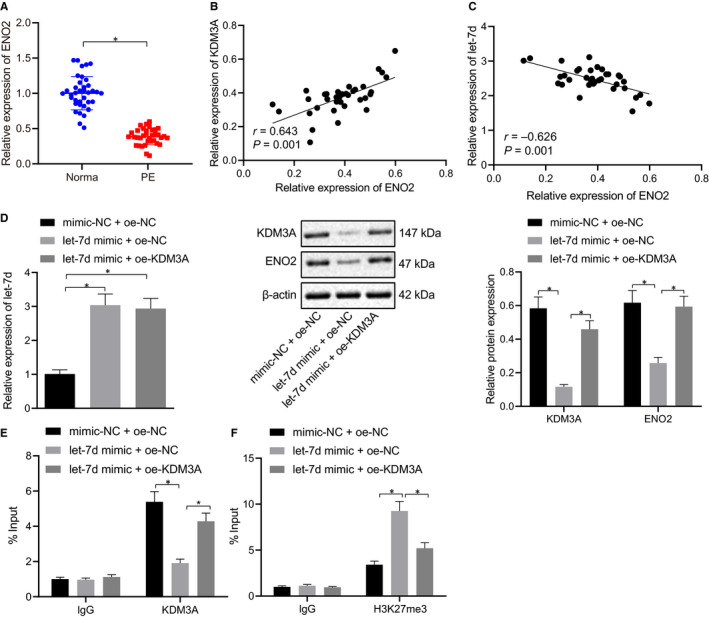
Let‐7d enhanced ENO2 histone methylation to inhibit ENO2 expression of via KDM3A. A, ENO2 expression in placental tissues in PE patients and healthy pregnant women was determined using RT‐qPCR, normalized to GAPDH. B, Correlation between ENO2 and KDM3A in placental tissues in PE patients was analysed. C, Correlation between ENO2 and let‐7d in placental tissues in PE patients was analysed. BeWo cells were treated with exogenous let‐7d mimic and/or expression vectors containing KDM3A. D, let‐7d expression and protein levels of KDM3A and ENO2 in BeWo cells were determined using RT‐qPCR and Western blot analysis, respectively. E, Binding of KDM3A and promoter of ENO2 gene was detected by ChIP assay. F, H3K27me3 levels at the promoter site of ENO2 gene were detected by ChIP assay.* (relative to BeWo cells treated with mimic‐NC + oe‐NC or IgG antibody) indicate *P* < .05. Unpaired t test was used to analyse data between two groups and one‐way ANOVA/Tukey's test to analysed data among multiple groups. The correlation among let‐7d, KDM3A and ENO2 was analysed using Pearson analysis

The results of the RT‐qPCR analysis revealed that the expression of ENO2 was decreased in the placental tissues of PE patients (*P* < .05) (Figure 5A). Additionally, Pearson correlation analysis demonstrated that the expression of ENO2 was positively correlated with KDM3A (Figure 5B) and negatively correlated with let‐7d (Figure 5C). Furthermore, the BeWo cells were transfected with exogenous the let‐7d mimic and/or expression vectors containing KDM3A. The RT‐qPCR and Western blot analyses of the transfected cells showed decreased expression of KDM3A and ENO2 in the BeWo cells treated with exogenous let‐7d mimic, and an increased ENO2 expression in BeWo cells which were treated with exogenous let‐7d mimic and expression vectors containing KDM3A (*P* < .05) (Figure 5D). On the basis of the abovementioned results, it was further speculated that let‐7d could down‐regulate the expression of KDM3A through the methylation of ENO2. Therefore, the ChIP assay primers were designed for the ENO2 promoter site. The results of the ChIP assay showed that the ENO2 gene promoter binding to the KDM3A antibody was increased, which suggested that the histone demethylase KDM3A could bind to the promoter region of the ENO2 gene. Furthermore, it was established that the overexpression of let‐7d inhibited the KDM3A and therefore subsequently reduced KDM3A enrichment in the ENO2 promoter (Figure 5E). Similarly, the overexpression of let‐7d enhanced the H3K27me3 enrichment in the ENO2 promoter; whereas, the subsequent KDM3A overexpression reduced the H3K27me3 enrichment (Figure 5F). Cumulatively, the aforementioned results confirmed that let‐7d affected the H3K27me3 levels at the promoter site of the ENO2 gene through KDM3A, thus inhibiting the expression of ENO2.

### 3.6 Let‐7d suppressed the proliferation, migration and invasion of trophoblasts and enhanced apoptosis by regulating ENO2 via KDM3A down‐regulation

**FIGURE 6:**
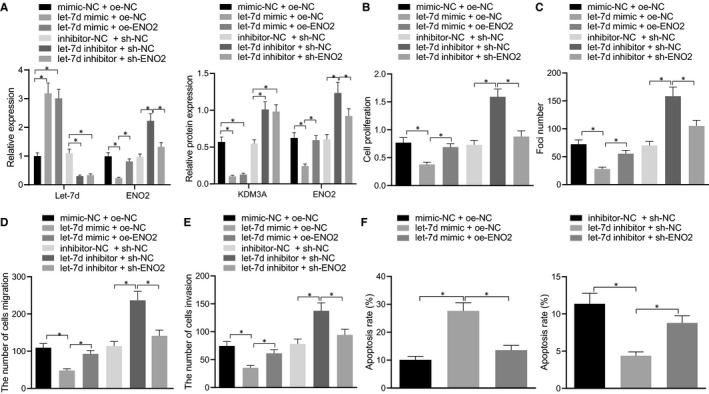
Let‐7d suppressed proliferation, migration, and invasion of trophoblasts and enhanced apoptosis by regulating ENO2 via down‐regulation of KDM3A. BeWo cells were treated with exogenous let‐7d mimic, exogenous let‐7d inhibitor, expression vectors containing ENO2 and short hairpin RNA targeting ENO2. A, Expression of let‐7d, KDM3A and ENO2 in BeWo cells was determined using RT‐qPCR and Western blot analysis. B, Proliferation of BeWo cells was detected by MTT assay. C, Clone formation of BeWo cells was detected by colony formation assay. D, Migration of BeWo cells was detected by Transwell assay. E, Invasion of BeWo cells was detected by Transwell assay. F, Apoptosis of BeWo cells detected by Flow cytometry. * (relative to BeWo cells treated with mimic‐NC + oe‐NC, let‐7d mimic + oe‐NC, inhibitor‐NC + sh‐NC or let‐7d inhibitor + sh‐NC) indicate *P* < .05 by one‐way ANOVA/Tukey's test

The BeWo cells were transfected with exogenous let‐7d mimic, exogenous let‐7d inhibitor, expression vectors containing ENO2, and shRNA targeting ENO2 to further explore whether let‐7d affected the cell proliferation, migration and apoptosis of human trophoblasts by regulating ENO2. The RT‐qPCR analysis was adopted to measure the let‐7d expression and the ENO2 mRNA levels; whereas, the Western blot analysis was employed to determine the KDM3A and ENO2 protein levels (Figure 6A). Additionally, the results of the MTT assay (Figure 6B), the colony formation assay (Figure 6C), the Transwell assay (Figure 6D, E) and the Flow cytometry (Figure 6F) cumulatively revealed that the cell proliferation, migration, and invasion were inhibited, the number of clone formation was decreased, and the apoptosis was promoted in the BeWo cells transfected with exogenous let‐7d mimic, or exogenous let‐7d inhibitor and the shRNA targeting ENO2. Meanwhile, the trends were found to be reversed in BeWo cells transfected with exogenous let‐7d inhibitor, or exogenous let‐7d mimic and expression vectors containing ENO2. Therefore, we concluded that let‐7d led to the inhibition of cell proliferation, migration and invasion of trophoblasts and facilitated the apoptosis by regulating the KDM3A‐mediated ENO2.

### 3.7 Silencing of let‐7d inhibited the occurrence of PE by up‐regulation of KDM3A‐mediated ENO2

**FIGURE 7:**
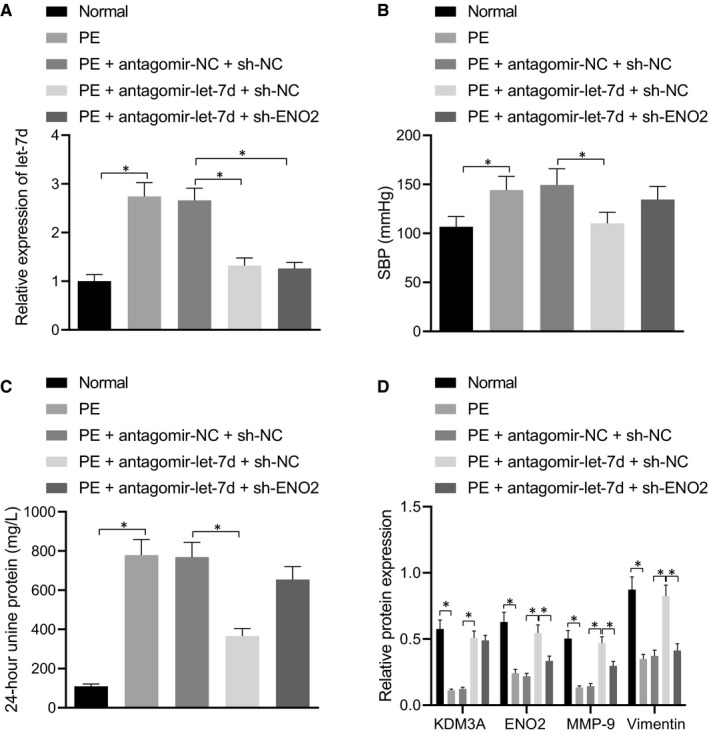
Down‐regulation of let‐7d suppressed the occurrence of PE by elevating ENO2 and KDM3A. PE rats were injected with antagomir‐NC + sh‐NC (n = 8), antagolet‐7d + sh‐NC (n = 8) and antagolet‐7d + sh‐ENO2 (n = 8). A, Let‐7d expression in placenta of healthy rats (n = 8) and PE rats was determined using RT‐qPCR. B, SBP of healthy rats and PE rats were measured. C, 24‐hour urine protein levels of healthy rats and PE rats were measured. D, Protein levels of KDM3A, ENO2, MMP‐9 and Vimentin in placental tissues of healthy rats and PE rats were determined using Western blot analysis, normalized to β‐actin. * (relative to healthy rats) indicate *P* < .05 by unpaired t test and # (relative to PE rats injected with antagomir‐NC + sh‐NC) indicate *P* < .05 by one‐way ANOVA/Tukey's test

A rat model of PE was established to further explore the therapeutic effect of let‐7d on PE by regulating ENO2. The PE rats were injected with antagomir‐NC + sh‐NC, antagolet‐7d + sh‐NC and antagolet‐7d + sh‐ENO2. The RT‐qPCR analysis revealed that the expression of let‐7d was elevated in the placentas of PE rats, while it decreased in PE rats injected with antagolet‐7d + sh‐NC or antagolet‐7d + sh‐ENO2 (Figure 7A). Additionally, the PE rats showed increased SBP and 24‐hour urine protein levels (Figure 7B, C). Furthermore, the HE staining of the rat placental tissues demonstrated placental villous cellular necrosis, increased trophoblast nodules, loss of vascular membranes and narrowed lumens (supplementary Figure 1A). Moreover, the immunohistochemical staining (supplementary Figure 1B), TUNEL staining (supplementary Figure 1C), Western blots (Figure 7D) cumulatively established the decreased expressions of KDM3A, ENO2, MMP‐9 and Vimentin and increased apoptosis. Consequently, the inhibition of let‐7d significantly reduced the SBP and the 24‐hour urine protein, alleviated the PE‐related tissue damage, and decreased amount of TUNEL‐positive cells. Whereas, the expression levels of KDM3A, ENO2, MMP‐9 and Vimentin in the placentas of the PE rats were revealed to be significantly increased. On the basis of the abovementioned results, the current study concluded that the effect of let‐7d could be weakened subsequent to the interference of ENO2.

## 4 DISCUSSION

PE is the leading cause of foetal mortality and maternal mortality during pregnancy, and accounts for 50 000 to 75 000 maternal deaths worldwide each year.^6^ The understanding of its pathobiology and diagnosis is still poor.^18^ The imbalance of trophoblast homeostasis plays an important role in the development of PE.^19^ Trophoblasts contribute to the formation of the placenta through the invasion into the myometrium and trigger the onset of PE, also amongst PE patients the notable inhibition of trophoblast proliferation, invasion and migration has been established.^20^ Recently, miRNAs have been considered to be a potential therapeutic biomarker of PE.^21^ However, the mechanism of let‐7d in PE remains elusive. Therefore, it is essential to investigate the underlying mechanism. Based on the results of the present study, it was concluded that overexpressed let‐7d targeted KDM3A via regulation of ENO2. By this mechanism, it suppressed proliferation, migration and invasion of trophoblasts, but enhanced apoptosis, thereby facilitating the progression of PE.

The data obtained in the current study demonstrated that let‐7d was highly expressed in PE, which suppressed cell proliferation, migration and invasion of trophoblasts and enhanced the apoptosis to subsequently stimulate the development of PE. Additionally, Brkić *et al* have also reported that the aberrant expression of miRNAs is implicated in trophoblast invasion and differentiation in the progression of PE.^4^ The various mechanisms of miR‐target gene interactions that are involved in the development of PE have been explored recently.^22^ For example, there is a potential correlation between miR‐210 and miR‐155 and the onset of PE since these 2 miRNAs have been found to be highly expressed in the serum of pregnant women with PE.^23^ Furthermore, miR‐515‐5p has been previously established to be up‐regulated in PE, and contributes to the pathogenesis of PE.^24^ The effects of miRNAs have been also validated in trophoblast cell migration and invasion and their roles in the progression of PE.^7^ Additionally, the expression of let‐7d was found to be increased in the PE placental tissues. Conversely, the down‐regulation of let‐7d contributes to the suppression of apoptosis and facilitation of proliferation and invasion of trophoblasts in PE.^25^ This suggests a key role of let‐7d in human trophoblast proliferation and invasion in PE. Cumulatively, there is significant evidence that let‐7d is able to promote the progression of PE by inhibiting the trophoblast proliferation, migration and invasion, and promoting apoptosis.

The present study also demonstrated that let‐7d could target and negatively regulate KDM3A, which was found to be highly expressed in placental tissues and trophoblasts in PE. KDM3A is a H3K9 demethylating enzyme, and demethylated H3K9 is known to increase transcriptional activity.^26^ Additionally, histone demethylase KDM3A could potentially regulate cell fate to strengthen the view that histone demethylases are essential to cell differentiation.^27^ The current study also proved that KDM3A promoted proliferation, migration and invasion of trophoblasts and inhibited apoptosis in PE. Furthermore, KDM3A has been reported to be involved in multiple biological activities in tumours.^9^ It is interesting to note that hypoxia could affect trophoblast differentiation and regulate placental organization through KDM3A.^10^ The role of the hypoxia‐inducible factor‐1alpha (HIF‐1α) has also been demonstrated in the pathogenesis of PE.^5^ Moreover, let‐7d was demonstrated to promote ENO2 histone methylation to inhibit ENO2 expression of through KDM3A. Another recent study has revealed that ENO2 exerts great effects on multiple human cancers.^12^ Tong et al have found that ENO2 expression was reduced in PE,^11^ while its underlying mechanism in PE remains to be further studied. According to the above mentioned findings, the current study suggested that ENO2 could inhibit the progression of PE. The results of the current study further demonstrated that the silencing of let‐7d functioned as a suppressor in PE by regulating cellular processes through the up‐regulation of ENO2 and KDM3A. In the present study, L‐NAME a nitric oxide synthase inhibitor was applied which causes vasomotor dysfunction, increases blood pressure and affects foetal development to induce animal models of PE. The nitric oxide synthase inhibition model mimics human pre‐epilepsy with similar symptoms and this model has the advantage of simple operation, good repeatability, and high success rate. It is also very similar to human pre‐epilepsy in terms of pathophysiology, and is an ideal animal model for studying pre‐epilepsy.^28^ However, the animal model constructed by this method also has limitations: L‐NAME, as an inhibitor of NOS, may affect foetal development and cause foetal malformations.^29^

**FIGURE 8:**
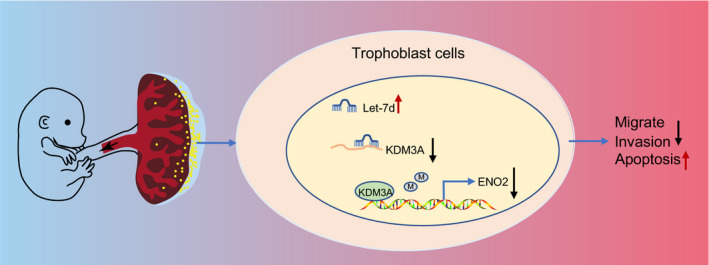
Underlying mechanisms concerning the role of let‐7d in PE. Let‐7d suppressed trophoblast proliferation, migration and invasion, and induced apoptosis by targeting KDM3A via the regulation of ENO2, leading to the facilitation of PE development

In conclusion, let‐7d suppressed trophoblast proliferation, migration and invasion, and induced apoptosis by targeting KDM3A via the regulation of ENO2, leading to the facilitation of PE development (Figure 8). Therefore, the identification of let‐7d may facilitate the existing understanding of the pathogenesis of PE, with a potential of serving as a diagnostic and prognostic marker for the treatment of PE in the future. Further studies are required, however, to fully understand the specific mechanisms of let‐7d targeting KDM3A via ENO2 in PE.

## CONFLICT OF INTEREST

The authors declare that they have no competing interests.

## AUTHOR CONTRIBUTION

**Qian Xu:** Conceptualization (equal); Investigation (equal); Supervision (equal); Writing‐original draft (supporting). **Yonghui Song:** Methodology (equal); Project administration (equal); Resources (equal). **Lili Lu:** Data curation (lead); Formal analysis (equal); Writing‐review & editing (equal).

## Supporting information

Fig S1

Table S1

## Data Availability

Research data are not shared.
