# Supplementary material for: Overexpression of let‐7d explains down‐regulated KDM3A and ENO2 in the pathogenesis of preeclampsia
Source: J Cell Mol Med. 2021 Aug 5;25(17):8127–39. doi: 10.1111/jcmm.16299 (PMC8419194; doi:10.1111/jcmm.16299)
Supplement: Supplementary file 2 — Table S1 [file JCMM-25-8127-s001.docx]

**Supplementary table 1** Clinical information of patients

| Group | Normal group | Preeclampsia group | | P valve |
| --- | --- | --- | --- | --- |
|  | Normal | Early preeclampsia | Severe preeclampsia |  |
| Number | 38 | 20 | 15 | *p*>0.05 |
| Age | 30.67±2.56 | 30.92±1.89 | 31.27±3.85 | **p*>0.05  #*p*>0.05  $ *p*>0.05 |
| Gestational age | 28.55±1.09 | 28.36±2.82 | 27.31±3.07 | **p*>0.05  #*p*>0.05  $ *p*>0.05 |
| Symptoms | SBP < 140 mmHg and DBP <  90 mmHg; without adverse reaction. | SBP ≥  140 mmHg and DBP ≥  90 mmHg with proteinuria of ≥  300 mg/day (or a protein/creatinine ratio of ≥  0.3 mg/dl or proteinuria of ≥  1+) or without proteinuria but with severe clinical features after 20 weeks of gestation with normal blood pressure | SBP (>160, >170,  >180 mmHg); DBP (>100; >110; >120mmHg ); proteinuria in g/l  (>2; >3; >5). |  |

*presented Normal group vs Early preeclampsia

# presented Normal group vs Severe preeclampsia

$ presented Early preeclampsia vs Severe preeclampsia

SBP, Systolic blood pressure; DBP, Diastolic blood pressure.
